# Global and regional burden of chikungunya from 2004 to 2024: a worldwide observational study

**DOI:** 10.7189/jogh.16.04055

**Published:** 2026-02-13

**Authors:** Sijia Wang, Yutong Liu, Yaping Wang, Liyan Zhou, Jue Liu

**Affiliations:** 1 Department of Epidemiology and Biostatistics, Peking University, Beijing, China; 2 Key Laboratory of Epidemiology of Major Diseases, Peking University, Beijing, China; 3 Institute for Global Health and Development, Peking University, Beijing, China

## Abstract

**Background:**

Chikungunya has emerged as a growing global health threat with a new sharp rise in outbreaks across 119 countries. However, its transmission patterns remain poorly characterised. We aimed to describe the global burden and spatiotemporal trends of chikungunya, and identify country-level environmental and socioeconomic factors associated with local transmission.

**Methods:**

We compiled annual country-level autochthonous chikungunya cases from 2004 to 2024, using data from regional surveillance systems and peer-reviewed sources. We calculated the incidence rates using the number of new cases and the population. We employed a generalised additive model (GAM) to flexibly model nonlinear associations between chikungunya incidence and environmental and socioeconomic factors. We performed subgroup analyses across the six WHO regions and conducted multiple sensitivity analyses addressing data structure, variable selection, and alternative model specifications to assess robustness.

**Results:**

Between 2004 and 2024, the global incidence of chikungunya from autochthonous cases rose from 0.28 to 11.13 per 100 000. In 2024, the Americas bore the heaviest burden (43.9 per 100 000; 431 305 cases), followed by South-East Asia (14.3 per 100 000; 258 854 cases), while Africa, Europe, and the Western Pacific reported few cases. Local outbreaks occurred recurrently in several countries across the Americas, South-East Asia, and Western Pacific, but remained sporadic in temperate regions. Using a GAM, we identified significant nonlinear effects of temperature, urbanisation, and GDP *per capita* on incidence: incidence rose sharply above 17°C; urban population percentage demonstrated a complex, nonlinear relationship; and GDP showed an inverse association at low to moderate levels. After adjusting for environmental and socioeconomic factors, the temporal trend of incidence generally declined. We identified notable heterogeneity across regions, while our results otherwise remained broadly consistent across the sensitivity analyses.

**Conclusions:**

Chikungunya burden has expanded globally, shaped by environmental and socioeconomic factors. Strengthened surveillance, integration of climate information into preparedness efforts, and improvements in socioeconomic conditions are needed to reduce disparities and support more effective prevention of future outbreaks.

Chikungunya fever is an acute mosquito-borne illness caused by the chikungunya virus (CHIKV), an alphavirus primarily transmitted by *Aedes aegypti* and *Aedes albopictus* mosquitoes [1]. Clinical manifestations typically include sudden-onset high fever, severe arthralgia, myalgia, headache, rash, and fatigue. Although life-threatening complications and fatalities are rare, arthritis-like pain can persist for months to years in some patients [2]. There is no specific antiviral therapy for chikungunya; current medical care is largely supportive, involving antipyretics and analgesics, fluids, and rest. Preventive strategies primarily focus on vector control, such as eliminating mosquito breeding sites, using repellents, wearing protective clothing, and avoiding bites. Two vaccines (live-attenuated and virus-like particle platforms) have recently received approval in the USA and EU; however, their limited availability restricts widespread immunisation [1].

Although chikungunya had been largely restricted to tropical Africa and Asia until the early 2000s, its global footprint has since expanded dramatically. A major epidemic beginning in coastal East Africa in 2004 triggered widespread transmission across the Indian Ocean islands and the Indian subcontinent [3,4]. Imported cases subsequently facilitated outbreaks in temperate regions such as southern Europe, demonstrating the virus’s capacity to establish local transmission where competent vectors are present. Autochthonous cases were first reported in the Americas in 2013, followed by sustained regional spread [4]. As of December 2024, 119 countries and territories have reported local transmission [5], and global modelling estimates suggest there are nearly 35 million new CHIKV infections annually [6].

Although CHIKV infection is typically self-limiting, it can lead to severe clinical manifestations and prolonged disability, particularly in vulnerable populations. Infants, older adults, and individuals with comorbidities are more likely to experience complications, including severe neurologic or systemic outcomes [1,3]. As global populations age, the long-term burden of post-chikungunya sequelae is expected to increase, further straining healthcare systems in affected regions.

Despite the growing geographic range and frequency of chikungunya outbreaks, comprehensive syntheses of chikungunya epidemiology remain limited. Much of the existing evidence derives from regional outbreak investigations or modelling studies, and cross-country comparisons are challenged by heterogeneous reporting systems [3,4,6]. These gaps impede accurate assessment of burden and hinder coordinated public health responses, especially as climatic and demographic changes are expected to alter transmission risk.

The transmission dynamics of CHIKV are influenced by an interaction between ecological, environmental, and socioeconomic factors. While prior work has examined environmental suitability using ecological niche models or geospatial tools [7–10], many analyses focus on vector presence rather than human cases, or are geographically restricted.

Beyond environmental factors, socioeconomic determinants such as income level, urban infrastructure, and development indices are increasingly recognised as critical drivers of arboviral outbreaks. Several localised studies have reported inverse associations between socioeconomic status and chikungunya incidence, although the indicators used vary widely, and findings remain context-dependent [11,12]. Notably, most previous modelling studies have examined CHIKV in conjunction with other arboviruses such as Zika or dengue, rather than as an independent focus of analysis. To address these challenges, we compiled country-year data on autochthonous cases from 2004–2024 and described global and regional patterns using standardised data sources. We then applied a generalised additive model (GAM) to explore nonlinear associations between climatic and socioeconomic factors and reported incidence within a consistent analytic framework.

## METHODS

### Study design and data source

We performed a multi-country observational analysis to assess the global and regional burden of chikungunya from 2004 to 2024. The research consists of two core components: a descriptive epidemiological assessment and an exploratory risk factor analysis.

We compiled country-year-level data on chikungunya case counts based on international surveillance systems, national health authorities, and global open-access databases. We derived case data from sources including the World Health Organization (WHO), European Centre for Disease Prevention and Control (ECDC), Pan American Health Organisation (PAHO), and Africa Centres for Disease Control and Prevention, supplemented them with high-quality peer-reviewed epidemiological reviews and verified grey literature, defined as official outbreak bulletins or epidemiological publications released by governmental or intergovernmental public health agencies.

For our covariates, we obtained mean annual temperature (°C) from the ERA5 reanalysis data set [13], and retrieved urban population percentage and country-level GDP *per capita* (USD) from the World Bank [14,15]. For sensitivity analyses, we additionally incorporated two development-related indicators. We sourced the sociodemographic index (SDI) from the GBD 2021 database [16]. The SDI acts as a composite indicator calculated by total fertility rate for women younger than 25 years, mean years of education for individuals aged 15 years and older, and lag distributed income *per capita* ranging from 0 to 1, which can reveal the economic development status relevant to health outcomes. We also included the percentage of people using at least basic sanitation services, obtained from the World Bank [17]. This indicator reflects access to improved sanitation facilities not shared with other households and includes both basic and safely managed services, such as flush or pour-flush systems connected to sewers or septic tanks and improved latrines. We matched all covariates to each country-year observation to ensure temporal alignment with the corresponding case data.

We applied specific inclusion and exclusion criteria to determine eligible data sources. We included data records if they were from official surveillance systems, government statistical yearbooks, outbreak bulletins, and peer-reviewed epidemiological reviews; and provided information on country, year, and the number of chikungunya cases. We excluded serological surveys, case reports, unverifiable media articles, and any records lacking information on geographic location, timing, or case definitions. Additionally, reports providing only estimates or covering imported cases without evidence of local transmission were omitted. We collected data from 119 countries with reported CHIKV transmission, as noted in WHO reports [5], of which 103 countries and 312 country-year observations met the criteria and were included in the final analysis.

To reduce inconsistencies arising from heterogeneous reporting systems, we conducted cross-source harmonisation and applied a predefined hierarchical rule in which we prioritised official national surveillance reports and WHO/PAHO epidemic updates when discrepancies occurred. While these procedures improve data consistency, they cannot fully eliminate underreporting, which remains an inherent limitation of global arboviral surveillance.

### Procedure

Our analysis included a total of 103 countries across six WHO regions from 2004 to 2024 (Table S1 in the **Online Supplementary Document**). We constructed a country-year panel data set, with each row representing a single country in a given year, comprising information on reported chikungunya cases, population size, mean annual temperature, urban population percentage, and GDP *per capita*.

We focused on autochthonous cases – locally acquired infections – since these better reflect endemic transmission dynamics and the environmental suitability for disease spread. If both ‛suspected’ and ‛confirmed’ cases were reported, we used the total number of suspected and confirmed cases unless specifically labelled otherwise. Where case data were separated into imported *vs.* local cases, we only retained autochthonous cases. In the rare instance where an outbreak spanned two calendar years, we allocated cases proportionally based on outbreak months and verified through sensitivity analyses that this approach did not materially affect the results. When monthly data were available, we summed all reported monthly case counts to obtain annual totals.

We calculated incidence rates (per 100 000 population) for each country-year by dividing the number of autochthonous cases by the total population, and obtained population data from the World Bank [18].

For temperature, we first aggregated monthly gridded temperature data to compute the mean annual temperature for each raster cell. We then spatially averaged these values at the country level, using country polygon boundaries, to derive a single national annual mean temperature for each year.

### Statistical analysis

We first performed descriptive analyses to characterise the temporal and regional patterns of chikungunya. We calculated the annual reported incidence (cases per 100 000 population) using the number of reported cases and corresponding population estimates. For both global and regional incidence estimates, we additionally calculated exact 95% confidence intervals using Poisson-based methods. We summarised regional trends by WHO region, and visualised incidence using line charts and choropleth heatmaps. In addition, to illustrate the spatial distribution and highlight differences in disease burden across countries, we also calculated the outbreak frequency for each country, defined as the number of years with at least one reported autochthonous case during the study period. We presented the results in the form of a global heatmap.

To explore the association of environmental and socioeconomic factors with CHIKV transmission, we employed a panel-data modelling approach based on count regression. We modelled the expected number of chikungunya cases *Y_ij_* in country *j* during year *i* using a GAM with a log link function. GAM’s flexibility allows it to capture nonlinear and non-monotonic relationships without restrictive assumptions, making it well-suited for the complex dynamics of vector-borne diseases. This approach enables a unified, chikungunya-specific analysis at a global scale, overcoming the limitations of fragmented, region-specific studies and providing a robust basis for targeted surveillance and control under changing climatic and socioeconomic conditions. The model is specified as:



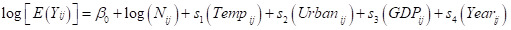



where E[Y*_ij_*] denotes the expected number of cases, and *N_ij_* is the population size of country *j* in year *i*, included as an offset to account for differing population sizes. The intercept is represented by *β*_0_, while *s_k_* (⋅) denotes smooth functions applied to each continuous covariate to flexibly capture potentially nonlinear relationships. Specifically, Temp*_ij_* corresponds to the mean annual temperature (°C), Urban*_ij_* is the urban population percentage (%), GDP*_ij_* is the gross domestic product *per capita* (USD), and Year*_ij_* represents the calendar year, all measured for country *j* in year *i*.

Given the tendency for socioeconomic indicators to exhibit structural dependence, we examined potential multicollinearity among smooth terms using concurvity indices – the appropriate diagnostic metric for GAMs. We considered values acceptable if the estimated concurvity was below the conventional threshold of 0.8. Because the determinants of CHIKV transmission may differ across ecological and socio-political environments, we also refitted the GAM separately within each of the six WHO regions to explore region-specific associations.

We conducted a comprehensive set of sensitivity analyses to rigorously assess robustness. First, we addressed the treatment of the single multi-year observation by excluding it; and allocating all cases to the first reported year. We then examined whether the observed socioeconomic patterns depended on the choice of development metric. We replaced GDP with the SDI, a composite indicator of societal development, and then with the percentage of people using at least basic sanitation services, a more proximate determinant of *Aedes* habitat suitability. In addition, we conducted stratified analyses by World Bank income level, fitting separate models for high-income and non-high-income countries to assess whether the GDP – incidence relationship was consistent across different development contexts.

Next, to account for potential dependence structures that could bias inference, we fitted three additional models: a first-order autoregressive (AR(1)) specification incorporating lag-1 cases to capture within-country temporal autocorrelation; a spatial GAM including a bivariate smooth over latitude and longitude to model spatial clustering; and a country-level random-effects GAM to account for unobserved, time-invariant heterogeneity.

Finally, to examine whether the smooth year term in the main model was capturing broader secular shifts, including the COVID-19-related disruption, we replaced the continuous year smooth with fixed-effect indicators for pre-pandemic (2004–2019), pandemic (2020–2021), and post-pandemic (2022–2024) periods. We evaluated model outputs from these alternative specifications relative to the main model based on the direction, significance, and functional form of key covariate effects. We conducted all statistical analyses using *R*, version 4.5.1. (R Core Team, Vienna, Austria). We considered a two-sided *P*-value less than 0.05 statistically significant.

## RESULTS

### Global and regional burden of chikungunya

Globally, the burden of autochthonous CHIKV infection has risen markedly over the past two decades. Among 103 countries in 2004, only the African Region reported autochthonous cases, resulting in a global incidence of 0.28 per 100 000 population. By contrast, the global incidence had increased to 11.13 per 100 000 population in 2024, with a total of 696 564 reported autochthonous cases (**Table 1**).

**Table 1:** Global and regional incidence and autochthonous chikungunya cases in 2004 and 2024*

Characteristics	Incidence per 100 000 population (95% CI) in 2004	Number of cases in 2004	Incidence per 100 000 population (95% CI) in 2024	Number of cases in 2024
Global	0.28 (0.27–0.28)	13 520	11.13 (11.10–11.15)	696 564
WHO Region				
*African Region*	2.34 (2.30–2.38)	13 520	0.002 (0.001–0.003)	18
*Eastern Mediterranean Region*	0.00 (0.00–0.00)	0	1.44 (1.40–1.48)	5726
*European Region*	0.00 (0.00–0.00)	0	0.01 (0.00–0.01)	10
*Region of the Americas*	0.00 (0.00–0.00)	0	43.90 (43.77–44.04)	431 305
*South-East Asia Region*	0.00 (0.00–0.00)	0	14.34 (14.29–14.40)	258 854
*Western Pacific Region*	0.00 (0.00–0.00)	0	0.03 (0.03–0.04)	651

CI – confidence interval, WHO – World Health Organization

*We calculated 95% CIs using exact Poisson methods. Values of ‛0.00’ indicate zero reported cases.

Across WHO regions (**Table 1**), the Region of the Americas experienced the most substantial chikungunya burden in 2024, reporting 431 305 cases and an incidence of 43.90 per 100 000 population. The South-East Asia Region followed with an incidence of 14.34 per 100 000 population and 258 854 cases, primarily driven by a large outbreak in India. The Eastern Mediterranean Region reported 5726 cases, corresponding to an incidence of 1.43 per 100 000 population. The Western Pacific Region reported 651 cases (0.03 per 100 000 population). In contrast, the African Region – which was historically associated with the emergence of CHIKV – reported only 18 cases in 2024, reflecting an incidence near zero. The European Region, previously considered non-endemic, also recorded 10 autochthonous cases (0.01 per 100 000 population).

### Temporal trends

**Figure 1:**
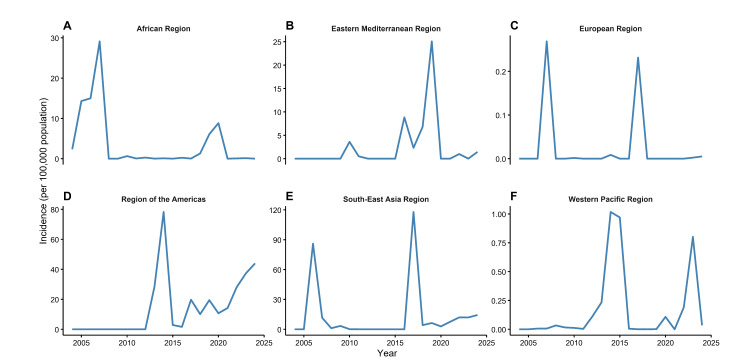
Incidence Trends of Chikungunya by WHO Region (2004–2024). **Panel A.** African Region. **Panel B.** Eastern Mediterranean Region. **Panel C.** European Region. **Panel D.** Region of the Americas. **Panel E.** South-East Asia Region. **Panel F.** Western Pacific Region. Although La Réunion and Mayotte are administratively part of France (WHO European Region), they were classified under the African Region in this analysis to better reflect the geographic distribution of CHIKV transmission patterns in each region.

From 2004 to 2024, the average reported incidence of chikungunya, based on autochthonous cases, showed marked temporal variation across WHO regions. The African Region experienced the highest peaks in 2006 and 2007, with incidences of 14.99 and 29.13 per 100 000 population, respectively, largely due to the outbreaks in Central Africa and the western Indian Ocean islands. In the Americas, incidence remained at zero before 2013, when autochthonous transmission was first reported in December on Saint Martin, followed by rapid spread to surrounding areas; incidence surged dramatically in 2014, reaching 78.21 per 100 000 population. The South-East Asia Region exhibited sporadic but intense peaks, with 86.00 per 100 000 population in 2006 and 117.87 per 100 000 population in 2017, mainly driven by outbreaks in India and Thailand. The Eastern Mediterranean Region reported no cases before 2010, followed by peaks in 2016 (8.83 per 100 000 population) and 2019 (25.07 per 100 000 population), with smaller resurgences in subsequent years. In contrast, the European and Western Pacific Regions reported very low incidences per 100 000 population, with occasional localised transmission events (**Figure 1**, Figure S1 in the **Online Supplementary Document**).

### Country-level outbreaks distribution

**Figure 2:**
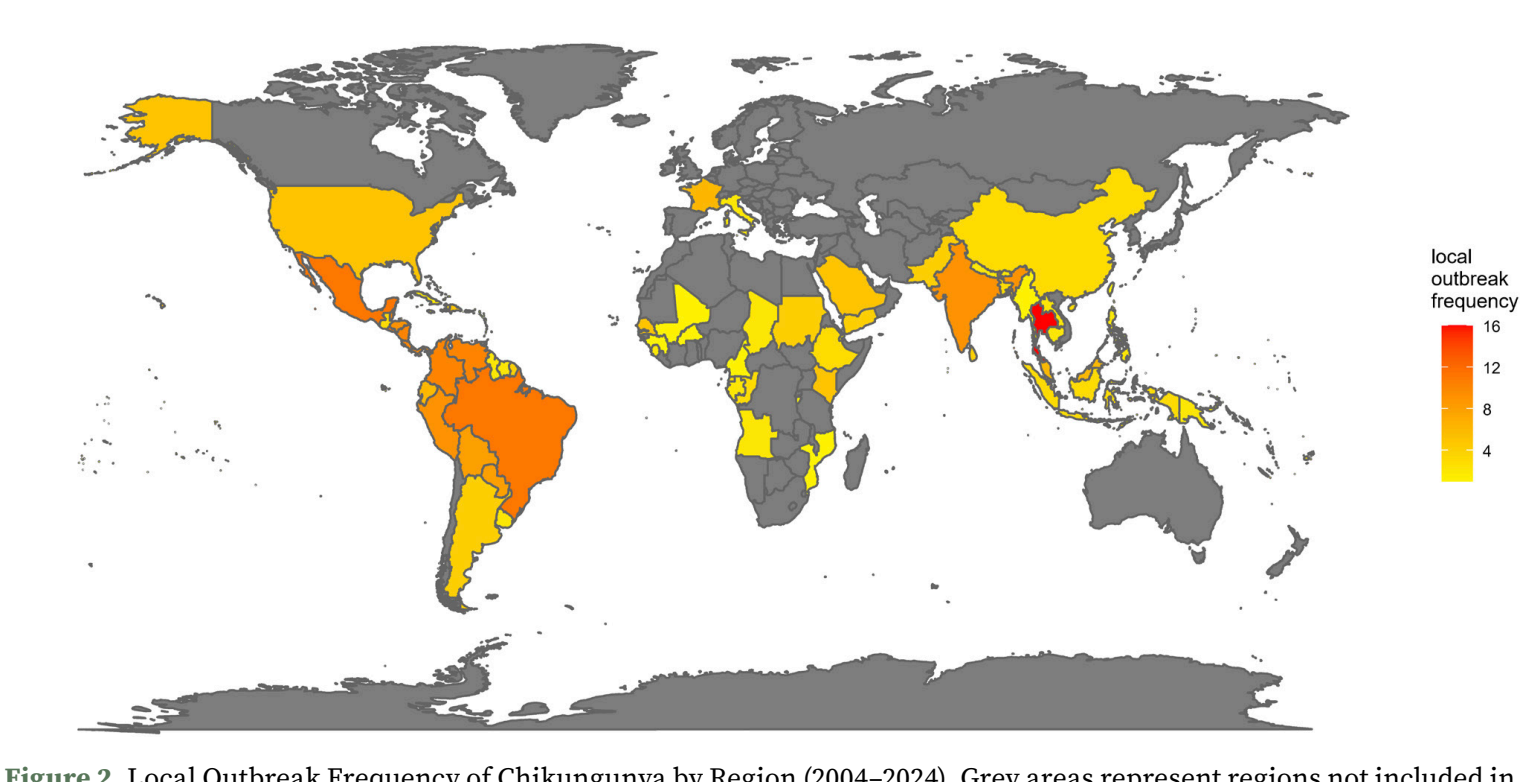
Local Outbreak Frequency of Chikungunya by Region (2004–2024). Grey areas represent regions not included in the study or with no available data.

The frequency of local chikungunya outbreaks varied widely across countries and regions from 2004 to 2024 (**Figure 2**). In the Southeast Asia and Western Pacific regions, countries such as Thailand (2008–2023), Singapore (2007–2020, 2022), and Malaysia (2006–2008, 2020, 2023–2024) experienced frequent outbreaks, reflecting persistent transmission in tropical and subtropical climates. The Region of the Americas showed widespread recurrent outbreaks, with Brazil, Mexico, El Salvador, Colombia, Panama, and Venezuela each reporting cases in most years following the virus’s introduction in 2013, and several Caribbean islands like Barbados (2014, 2018–2021, 2023–2024), Saint Lucia (2014, 2018, 2021), and the Virgin Islands (2013–2014) reporting multiple outbreak years. In the African Region, outbreak frequency was generally lower and more scattered, with countries such as Angola (2017–2018) and Cameroon (2006) reporting sporadic outbreaks. The European Region had very few outbreak years, with limited occurrences in countries including Italy (2007, 2017) and France (2010, 2014, 2017, 2023–2024). Similarly, the Eastern Mediterranean Region showed sporadic outbreaks in countries such as Saudi Arabia (2011, 2013, 2018, 2019, 2021) and Djibouti (2019; Table S1 in the **Online Supplementary Document**).

### Association analysis

We included 312 country-year observations from 103 countries spanning 2004 to 2024. The median number of autochthonous cases was 254 (interquartile range (IQR) = 2428), with a median incidence of 2.35 per 100 000 population (IQR = 35.63). The median population size was approximately 10.55 million (IQR = 43.77 million). Median temperature was 25.19°C (IQR = 2.58), and median urban population percentage was 62.66% (IQR = 36.11). The median GDP *per capita* was USD 6303.92 (IQR = 10 102.97), and the median SDI was 0.63 (IQR = 0.15) (**Table 2**).

**Table 2:** Summary of study sample characteristics from 2004 to 2024 (n = 312 country-year observations from 103 countries).

Variable	MD (IQR)
Autochthonous cases	254 (2428)
Incidence (per 100 000 population)	2.35 (35.63)
Population	10 551 430 (43 771 680)
Mean annual temperature in °C	25.19 (2.58)
Urban population percentage in %)	62.66 (36.11)
GDP in USD	6303.92 (10 102.97)
SDI	0.63 (0.15)

GDP – gross domestic product, IQR – interquartile range, MD – median, SDI – sociodemographic index

**Figure 3:**
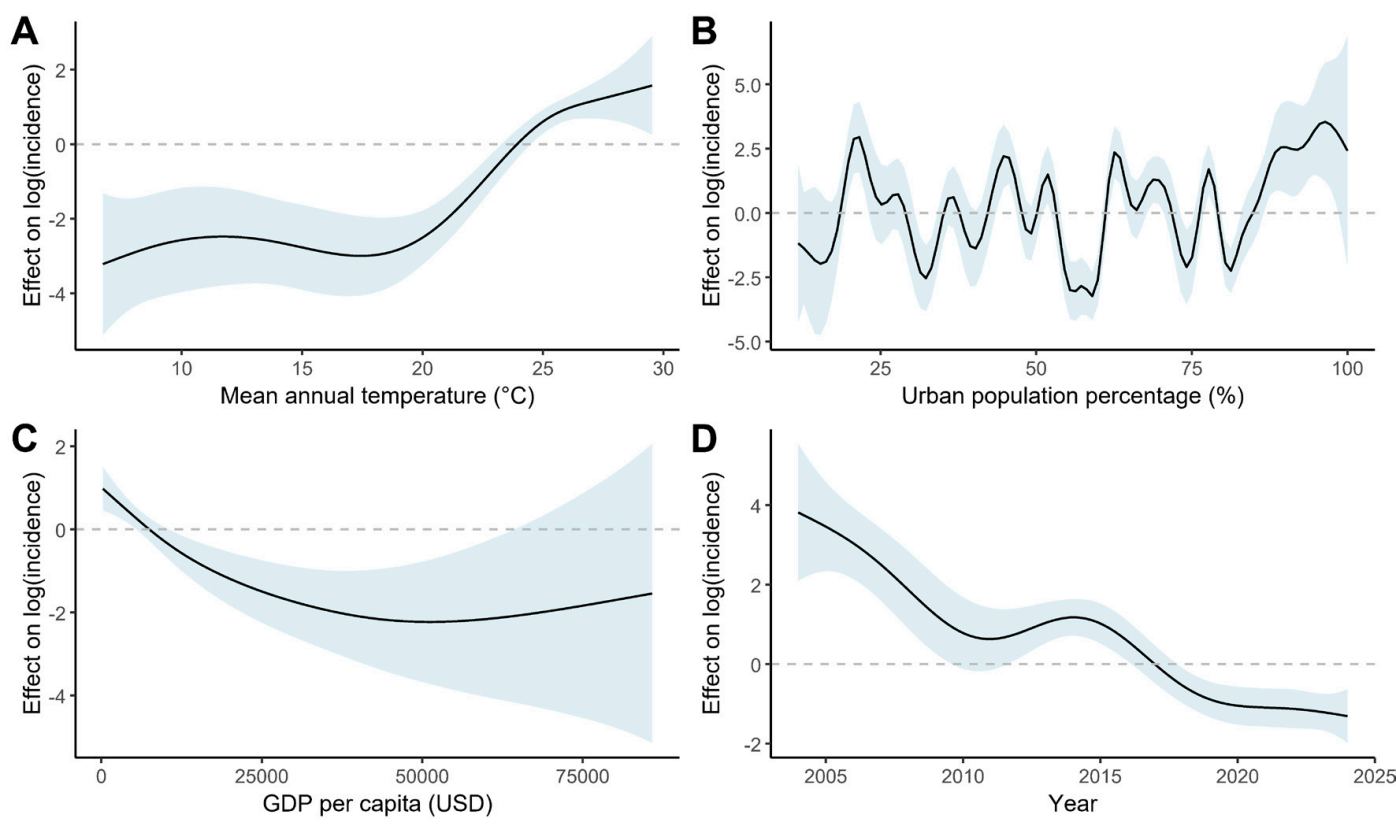
Partial Residual Plots Showing the Effects of Covariates on Chikungunya Incidence (Link Scale). **Panel A.** Mean annual temperature. **Panel B.** Urban population percentage. **Panel C.** GDP *per capita*. **Panel D.** Year. Shaded areas represent 95% confidence intervals.

We employed a GAM to explore the nonlinear associations between environmental and socioeconomic factors and the log-transformed incidence of chikungunya, with the final model explaining 57.1% of the deviance. Concurvity among smooth terms remained below 0.80, indicating no problematic functional dependencies. Mean annual temperature (estimated degrees of freedom (edf) = 4.46, *P* < 0.001) displayed a right-skewed J-shaped relationship with incidence, with a protective effect at ~8–17°C but rose sharply into the positive range at higher temperatures (>17°C), indicating a positive association with incidence. Urban population percentage (edf = 27.36, *P* < 0.001) showed highly irregular fluctuations up to ~85%, with no consistent positive or negative pattern; above ~85%, the effect was consistently positive, with risk increasing initially and then decreasing as urban population percentage approached the maximum. GDP *per capita* (edf = 2.69, *P* = 0.00012) exhibited a U-shaped association with chikungunya incidence, with risk decreasing as GDP increased from low to moderate levels. Finally, after adjusting for other variables, the temporal trend (Year; edf = 5.55, *P* < 0.001) showed an overall decrease in incidence, with a temporary increase around 2010 that peaked between 2014 and 2015, followed by a sustained decrease thereafter (**Figure 3**, **Table 3**).

**Table 3:** Generalised additive model results (n = 312 country-year observations from 103 countries)

Variables	Estimated degrees of freedom	X^2^	*P*-value
Mean annual temperature in °C	4.46	96.65	<0.001
Urban population percentage in %	27.36	245.68	<0.001
GDP in USD	2.69	22.10	0.00012
Year	5.55	105.32	<0.001

GDP – gross domestic product

In subgroup analyses by WHO region, all four variables showed statistically significant associations with incidence in the Region of the Americas and the African Region (Figure S2 and Table S2 in the **Online Supplementary Document**). In South-East Asia, three variables were significant, while temperature reached significance only at the 0.1 level. In Europe, only the urban population percentage shows a significant effect. In the Eastern Mediterranean, the urban population percentage and GDP were significantly associated with incidence.

Across all alternative data-handling strategies, the effects of temperature, urbanisation, and the temporal trend remained significant and preserved their overall functional forms (Figures S3 and S4; Tables S3–12 in the **Online Supplementary Document**). Excluding the cross-year observation and assigning all cases to the first reporting year yielded nearly identical estimates. Replacing GDP with SDI or sanitation coverage yielded similar patterns for the remaining covariates. Stratified analyses by World Bank income level showed that the negative association between GDP and incidence remained directionally consistent in both high-income and non-high-income settings. Models accounting for potential dependence structures, including AR(1) lag terms, spatial smoothing, and country-level random effects, produced stable associations. Replacing the smooth year term with fixed-effect pandemic periods produced consistent results: relative to the pre-pandemic period, the adjusted incidence was approximately 80% lower during the pandemic (incidence rate ratio (IRR) = 0.20, *P* < 0.001) and 76% lower in the post-pandemic period (IRR = 0.24, *P* < 0.001). Overall, the sensitivity analyses consistently supported the robustness of the primary GAM results.

## DISCUSSION

Our descriptive analysis reveals a sharp global rise in chikungunya incidence over the past two decades. The Americas and South-East Asia now bear the heaviest burdens of endemic CHIKV transmission, while Europe and the Western Pacific remain largely unaffected. Notably, the African Region reported very few cases in recent years, though this likely reflects underreporting due to limited surveillance capacity rather than the true absence of transmission [4]. Consequently, low recorded incidence in data-sparse settings should not be taken as indicative of low underlying disease burden. At the country level, outbreaks were concentrated in tropical regions, with frequent transmission reported in Brazil, India, Thailand, and several Central American countries. In contrast, most temperate and higher-income regions saw either no or sporadic local transmission. These patterns suggest that chikungunya burden is shaped not only by ecological suitability but also by differences in socioeconomic factors, explored further in our modelling analysis. In addition to these factors, part of the observed regional heterogeneity may also reflect variation in surveillance system characteristics, including case definitions, diagnostic availability, and reporting frequency, which influence the completeness and comparability of reported incidence across settings [5,19].

The spread of CHIKV is influenced by a range of environmental, social, and economic factors. First, across the main analysis, subgroup analyses, and sensitivity analyses, we consistently found a positive association between temperature and chikungunya incidence. This effect was particularly pronounced at temperatures above 25°C, where the curve lies above zero, indicating a positive effect (**Figure 3**). These findings are consistent with the known biological mechanisms by which temperature affects mosquito survival, reproduction, and viral development within the vector. Prior studies have shown that higher temperatures shorten the extrinsic incubation period of CHIKV within *Aedes* mosquitoes and enhance their biting frequency, thereby accelerating transmission [8,20]. Furthermore, climate change and global warming are expected to further elevate the risk of vector-borne diseases, including chikungunya, by expanding the geographical range and seasonality of vector activity [20].

Second, the relationship between urban population percentage and chikungunya incidence was highly nonlinear. No clear pattern emerged at lower levels, although we observed a dip between 50–60% urban population, corresponding to a negative association. Above approximately 85%, incidence increased with urban population percentage, and the association became positive (**Figure 3**). This heterogeneity likely reflects the composite nature of national-level urbanisation, which encompasses highly diverse forms of urban growth across settings and therefore may not represent specific transmission-relevant conditions. In higher-income countries with well-developed infrastructure, urbanisation may reduce exposure to *Aedes* mosquitoes, whereas in low-income contexts, rapid and unplanned urban growth often exacerbates risk by creating favourable breeding environments for mosquitoes. Subgroup analyses suggested such contextual differences, although estimates were unstable due to limited sample sizes. These findings underscore that urbanisation at the national level is an imprecise indicator of the underlying mechanisms linking built environments with arboviral risk. Future research should incorporate more proximate determinants, such as sanitation access, water storage practices, waste management systems, and housing quality, to better clarify the pathways through which urban development shapes chikungunya transmission.

Third, GDP showed a downward trend with incidence in the main model: lower GDP was associated with higher incidence while higher GDP appeared protective. This broad pattern is consistent with evidence from other *Aedes*-borne infections, where lower socioeconomic status has been linked to increased vector abundance and infection risk [21,22]. Higher GDP may correspond to improvements in housing quality and sanitation infrastructure that reduce vector breeding sites and human-vector contact [23]. Sensitivity analyses stratified by income category further supported the overall negative GDP-incidence association, as both high-income and non-high-income groups showed a declining risk with increasing GDP across the primary range of observed values. However, as GDP represents a distal socioeconomic proxy rather than specific determinants, its interpretation should remain cautious. In both the main model and the stratified analyses, we also observed a slight rise or flattening at very high GDP levels; given the small number of country-years in this range, this pattern is likely attributable to sparse data rather than a true epidemiological effect.

In the subgroup analysis, regional patterns were heterogeneous. While a protective association of GDP appeared in Europe, South-East Asia, and the Eastern Mediterranean, we observed the opposite pattern in the Americas, the Western Pacific, and Africa (Figure S2 in the **Online Supplementary Document**). These differences likely reflect variations in development trajectories, urban growth, and health system capacity across regions [24]. However, the number of country-year observations was limited in several regions, particularly Europe and the Eastern Mediterranean, which may have contributed to unstable estimates.

Finally, although descriptive analyses showed an overall increase in chikungunya incidence from 2004 to 2024, the GAM results revealed a declining temporal trend after adjusting for climate, socioeconomic status, and urbanisation. This divergence suggests that the apparent rise in raw incidence was largely driven by the rapid progression of these environmental and sociodemographic risk factors rather than time itself. Once these drivers are controlled for, the residual declining trend likely reflects background improvements in vector control strategies, public health infrastructure, or gradual shifts in population immunity [25]. To further examine these temporal dynamics and address potential secular shocks, we performed a sensitivity analysis replacing the continuous year term with categorical COVID-19 period indicators (pre-pandemic 2004–2019, pandemic 2020–2021, and post-pandemic 2022–2024). The results confirmed that, relative to the pre-pandemic era, the adjusted incidence of reporting cases was significantly lower during and after the COVID-19 pandemic, while the effects of key drivers (temperature, urbanisation, and GDP) remained robust (Table S12 in the **Online Supplementary Document**). This suggests that the composite temporal effect estimated in the main model partially captures major period-specific disruptions, such as the redirection of surveillance resources towards COVID-19 or the impact of mobility restrictions on transmission [26–28]. However, as the time effect in the model aggregates the influence of multiple unmeasured or unobservable processes, ranging from surveillance capacity to reporting biases, it should be interpreted with caution.

This study has several limitations. First, global surveillance data for chikungunya are incomplete and likely underreported due to nonspecific clinical symptoms shared with dengue and Zika, limited diagnostic capacity, and frequent co-infections that complicate case identification [29,30]. Second, reporting practices vary across countries and years, with some failing to distinguish between imported and autochthonous cases, introducing exposure misclassification and potential bias [30]. Third, data coverage is uneven, and the overall sample size is limited, particularly in regions such as Africa and parts of South-East Asia, where surveillance infrastructure is weak and standardised diagnostic protocols are often lacking [3,4]. In addition, subgroup analyses in some regions were based on relatively small numbers of observations and may therefore yield unstable estimates. These gaps constrain the representativeness and generalisability of our findings. Future research with more complete and systematically collected data are needed to strengthen the evidence base for chikungunya epidemiology and control. Fourth, the socioeconomic indicators used in this study, including GDP and urban population percentage, are indirect proxies that do not fully reflect household-level determinants such as water storage practices, housing quality, or vector control coverage, which limits causal interpretability. Lastly, our modelling focused exclusively on autochthonous cases to minimise bias from imported infections. As imported cases have been key drivers of outbreaks in several regions, future studies should incorporate travel-related transmission dynamics to enhance model comprehensiveness.

To effectively mitigate the global threat of chikungunya, several coordinated strategies are needed. First, strengthening routine surveillance, particularly in regions with sparse or inconsistent reporting, would improve burden estimation and support more timely detection of local transmission [31]. Second, given the robust association between higher temperatures and increased incidence, integrating basic environmental and climate information into existing surveillance systems may help anticipate periods of elevated risk [32,33]. Finally, the negative relationship between GDP and incidence suggests that improvements in broader socioeconomic conditions, including sanitation access, housing quality, and essential public services, may help reduce population vulnerability to chikungunya transmission [10,34,35].

## CONCLUSIONS

We described global patterns of chikungunya incidence and examines ecological and socioeconomic factors associated with transmission. While data limitations require cautious interpretation, the analysis suggests that chikungunya continues to impose a notable health burden in several regions, particularly the Americas and South-East Asia. The observed nonlinear associations indicate that incidence increased at higher temperatures, urban population percentage showed no consistent pattern across settings, and GDP was generally inversely associated with incidence. Our findings indicate the value of strengthening routine surveillance, improving integration of basic environmental and climate information into monitoring activities, and supporting socioeconomic conditions related to housing, sanitation, and essential public services to reduce population vulnerability to chikungunya. Future work should further refine regional assessments and incorporate more detailed indicators to better characterise underlying mechanisms.

## Additional material


Online Supplementary Document
