# Supplementary material for: Global and regional burden of chikungunya from 2004 to 2024: a worldwide observational study
Source: J Glob Health. 2026 Feb 13;16:04055. doi: 10.7189/jogh.16.04055 (PMC12902706; doi:10.7189/jogh.16.04055)
Supplement: Online Supplementary Document [file jogh-16-04055-s001.pdf]

Supplement to: Wang S, Liu Y, Wang Y, Zhou L, Liu J. Global and regional burden of chikungunya from 2004 to 2024: a worldwide observational study. J Glob Health. 2026;16:04055.

Table S1. List of countries included in the analysis, cumulative case count, and corresponding WHO regions (2004–2024)

Country	Region	Cumulative number of cases	Years with Cases
India	South-East Asia Region	2,368,701	2006, 2007, 2018, 2019, 2020, 2021, 2022, 2023, 2024
Bangladesh	South-East Asia Region	2,000,144	2014, 2017, 2024
Brazil	Region of the Americas	1,632,183	2013, 2014, 2016, 2017, 2018, 2019, 2020, 2021, 2022, 2023, 2024
Dominican Republic	Region of the Americas	308,379	2013, 2014, 2016, 2023, 2024
Comoros	African Region	215,001	2005, 2006, 2007
Paraguay	Region of the Americas	149,131	2017, 2018, 2019, 2020, 2021, 2022, 2023, 2024
El Salvador	Region of the Americas	143,449	2013, 2014, 2016, 2017, 2018, 2019, 2020, 2021, 2022, 2023, 2024
Guadeloupe	Region of the Americas	105,628	2013, 2014
Martinique	Region of the Americas	103,269	2013, 2014, 2017
Colombia	Region of the Americas	93,816	2013, 2014, 2017, 2018, 2019, 2020, 2021, 2022, 2023, 2024
La Réunion (France)	Region of the Americas	91,387	2005, 2006, 2007, 2023
Thailand	South-East Asia Region	77,623	2008, 2009, 2010, 2011, 2012, 2013, 2014, 2015, 2016, 2017, 2018, 2019, 2020, 2021, 2022, 2023
Sudan	Eastern Mediterranean Region	76,514	2018, 2019, 2022
Haiti	Region of the Americas	71,089	2013, 2014, 2018, 2019
Chad	African Region	65,540	2020
Ethiopia	African Region	55,219	2019, 2020, 2022
Nepal	South-East Asia Region	53,954	2013, 2022
Sri Lanka	South-East Asia Region	51,690	2006, 2007, 2008, 2024
Venezuela	Region of the Americas	44,169	2013, 2014, 2016, 2017, 2018, 2019, 2020, 2021, 2022, 2023
Pakistan	Eastern Mediterranean Region	43,744	2016, 2017, 2023, 2024
Djibouti	Eastern Mediterranean Region	41,162	2019
Puerto Rico	Region of the Americas	40,579	2014, 2015, 2017
Guatemala	Region of the Americas	27,891	2013, 2014, 2017
Gabon	African Region	24,287	2007, 2010

French Guiana	Region of the Americas	21,950	2013, 2014, 2015, 2017
Congo	African Region	21,817	2011, 2018, 2019
Kiribati	Western Pacific Region	18,655	2014, 2015
Mauritius	African Region	17,500	2005, 2006, 2007
Kenya	African Region	16,036	2004, 2016, 2017, 2018, 2022
Yemen	Eastern Mediterranean Region	15,270	2010, 2011, 2012, 2022
Maldives	South-East Asia Region	14,004	2006, 2007, 2019, 2024
Seychelles	African Region	13,450	2005, 2006
Mexico	Region of the Americas	12,273	2014, 2015, 2016, 2017, 2018, 2019, 2020, 2021, 2022, 2023
Bolivia	Region of the Americas	11,336	2017, 2018, 2019, 2020, 2021, 2022, 2023, 2024
Belize	Region of the Americas	9,695	2013, 2014, 2016, 2018, 2019, 2020, 2021, 2022, 2023, 2024
Indonesia	Western Pacific Region	9,594	2022, 2023, 2024
Saint Martin	Region of the Americas	9,445	2013, 2014, 2017
China	Western Pacific Region	6,223	2010, 2019, 2023
Honduras	Region of the Americas	5,883	2013, 2014, 2018, 2019, 2020, 2021, 2022, 2023, 2024
Nicaragua	Region of the Americas	5,568	2013, 2014, 2017, 2018, 2019, 2020, 2021, 2022, 2023, 2024
Argentina	Region of the Americas	5,445	2015, 2016, 2023, 2024
Samoa	Western Pacific Region	4,524	2014
Peru	Region of the Americas	4,360	2016, 2017, 2018, 2019, 2020, 2021, 2022, 2023, 2024
Dominica	Region of the Americas	3,763	2013, 2014
Philippines	Western Pacific Region	3,489	2022, 2023
French Polynesia	Western Pacific Region	3,359	2014, 2015
Panama	Region of the Americas	3,329	2013, 2014, 2017, 2018, 2019, 2020, 2021, 2022, 2023, 2024
American Samoa	Western Pacific Region	3,200	2014
Papua New Guinea	Western Pacific Region	3,180	2012, 2013

Grenada	Region of the Americas	3,098	2014, 2024
Tonga	Western Pacific Region	3,000	2014
Saint Vincent and the Grenadines	Region of the Americas	2,792	2014, 2016
Malaysia	Western Pacific Region	2,491	2006, 2007, 2008, 2020, 2023, 2024
Singapore	Western Pacific Region	2,013	2007, 2008, 2009, 2010, 2011, 2012, 2013, 2014, 2015, 2016, 2017, 2018, 2019, 2020, 2022
Barbados	Region of the Americas	1,934	2014, 2018, 2019, 2020, 2021, 2023, 2024
Micronesia	Western Pacific Region	1,761	2013, 2014
United States Virgin Islands	Region of the Americas	1,701	2014
Guinea	African Region	1,590	2012
Jamaica	Region of the Americas	1,526	2014, 2018, 2019
Marshall Islands	Western Pacific Region	1,317	2015
Cook Islands	Western Pacific Region	1,295	2014, 2015
Suriname	Region of the Americas	1,212	2014, 2019
Costa Rica	Region of the Americas	1,107	2013, 2014, 2017, 2018, 2019, 2020, 2021, 2022, 2023, 2024
Trinidad and Tobago	Region of the Americas	932	2014, 2015, 2016
Mayotte (France)	Region of the Americas	924	2006
Saint Lucia	Region of the Americas	886	2014, 2018, 2021
Saint Kitts and Nevis	Region of the Americas	657	2014, 2018
Burundi	African Region	646	2014
Anguilla	Region of the Americas	612	2014, 2015
Italy	European Region	607	2007, 2017
Senegal	African Region	592	2015, 2023, 2024
United States of America	Region of the Americas	491	2014, 2016, 2017, 2018, 2024
Aruba	Region of the Americas	483	2014
Burkina Faso	African Region	473	2023
Sint Maarten	Region of the Americas	470	2013, 2014

Cameroon	African Region	400	2006
Sierra Leone	African Region	400	2012, 2013
British Virgin Islands	Region of the Americas	394	2013, 2014
Bhutan	South-East Asia Region	232	2011, 2012
Ecuador	Region of the Americas	231	2013, 2014, 2017, 2018, 2019, 2020
Cambodia	Western Pacific Region	214	2011, 2012
Lao People's Democratic Republic	Western Pacific Region	211	2012, 2020
Cayman Islands	Region of the Americas	205	2014
Tokelau	Western Pacific Region	200	2014
Timor-Leste	South-East Asia Region	196	2023, 2024
Bahamas	Region of the Americas	178	2014, 2015, 2016
Uruguay	Region of the Americas	137	2019, 2023
New Caledonia	Western Pacific Region	123	2011, 2013, 2014, 2016
Montserrat	Region of the Americas	119	2014
Angola	African Region	102	2017, 2018
Fiji	Western Pacific Region	92	2015, 2016, 2017
Guyana	Region of the Americas	78	2014
Equatorial Guinea	African Region	60	2004, 2005, 2006
France	European Region	43	2010, 2014, 2017, 2023, 2024
Turks and Caicos Islands	Region of the Americas	41	2014, 2016
Saint Barthélemy	Region of the Americas	34	2013, 2017
Antigua and Barbuda	Region of the Americas	33	2014
Myanmar	South-East Asia Region	18	2019
Cuba	Region of the Americas	14	2014, 2015
Mali	African Region	8	2023
Mozambique	African Region	7	2013

Saudi Arabia	Eastern Mediterranean Region	6	2011, 2013, 2018, 2019, 2021
Gambia	African Region	1	2023

Table S2. Subgroup generalised additive model analysis by WHO region

Region	n, deviance explained (%)	Estimated degrees of freedom	χ^2	*P*-value
Region of the Americas	173 (57.80)			
Mean annual temperature in °C		1.00	52.41	<0.001
Urban population percentage in %		17.52	173.82	<0.001
GDP in USD		2.93	10.97	0.023
Year		3.01	41.23	<0.001
African Region	42 (80.10)			
Mean annual temperature in °C		1.00	32.01	<0.001
Urban population percentage		8.03	137.62	<0.001
GDP in USD		1.64	12.85	0.0018
Year		1.00	118.78	<0.001
Western Pacific Region	32 (74.30)			
Mean annual temperature in °C		3.24	48.74	<0.001
Urban population percentage		1.00	5.68	0.017
GDP in USD		1.00	5.29	0.021
Year		3.62	29.27	<0.001
South-East Asia Region	39 (60.00)			
Mean annual temperature in °C		0.019	9.07	0.065
Urban population percentage in %		1.00	5.45	0.020
GDP in USD		4.69	47.20	<0.001
Year		1.00	5.42	0.020
European Region	8 (94.90)			
Mean annual temperature in °C		1.62	3.74	0.22
Urban population percentage in %		1.00	4.29	0.038
GDP in USD		1.00	0.75	0.39
Year		1.00	0.00	0.98
Eastern Mediterranean region	18 (85.70)			
Mean annual temperature in °C		1.64	1.81	0.36
Urban population percentage in %		1.00	4.71	0.030
GDP in USD		1.02	30.03	<0.001
Year		1.00	2.23	0.14

GDP – gross domestic product

Table S3. Sensitivity analysis 1: Excluding the cross-year observation

Variables	Estimated degrees of freedom	X2	P

Mean annual temperature (°C)	4.73	101.57	<0.001
Urban population percentage (%)	27.71	257.51	<0.001
GDP (current US dollars)	2.59	17.07	<0.001
Year	1.01	83.66	<0.001

Table S4. Sensitivity analysis 2: Assigning all multi-year cases to the first reporting year

Variables	Estimated degrees of freedom	X2	P
Mean annual temperature (°C)	4.84	103.91	<0.001
Urban population percentage (%)	27.91	255.87	<0.001
GDP (current US dollars)	2.45	15.96	0.0013
Year	1.01	78.39	<0.001

Table S5. Sensitivity analysis 3: Replacing GDP with SDI

Variables	Estimated degrees of freedom	X2	P
Mean annual temperature (°C)	2.98	105.30	<0.001
Urban population percentage (%)	11.92	65.50	<0.001
SDI (Sociodemographic Index)	3.55	38.82	<0.001
Year	1.00	67.32	<0.001

Table S6. Sensitivity analysis 4: Replacing GDP with people using at least basic sanitation services (% of population)

Variables	Estimated degrees of freedom	X2	P
Mean annual temperature (°C)	5.38	134.67	<0.001
Urban population percentage (%)	26.94	257.76	<0.001
People using at least basic sanitation services (% of population)	3.35	35.20	<0.001
Year	2.22	57.38	<0.001

Table S7. Sensitivity analysis 5: GAM stratified by income level (High-income countries)

Variables	Estimated degrees of freedom	X2	P
Mean annual temperature (°C)	1.00	28.89	<0.001
GDP (current US dollars)	4.24	18.39	0.0032
Year	1.00	61.99	<0.001

Note: Urban population percentage (%) was removed because of concurvity.

Table S8. Sensitivity analysis 6: GAM stratified by income level (Non-high-income countries)

Variables	Estimated degrees of freedom	X2	P
Mean annual temperature (°C)	6.69	130.28	<0.001
GDP (current US dollars)	2.59	14.91	0.0025
Year	1.70	49.97	<0.001

Note: Urban population percentage (%) was removed because of concurvity.

Table S9. Sensitivity analysis 7: GAM with AR(1) temporal autocorrelation

Variables	Estimated degrees of freedom	X2	P
Mean annual temperature (°C)	5.52	30.77	<0.001
Urban population percentage (%)	17.66	140.22	<0.001
GDP (current US dollars)	1.49	5.97	0.032
Year	6.43	92.81	<0.001
AR(1) term: log(cases_lag1)	—	—	<0.001

Table S10. Sensitivity analysis 8: GAM with a spatial smooth term

Variables	Estimated degrees of freedom	X2	P
Mean annual temperature (°C)	5.17	70.49	<0.001
Urban population percentage (%)	12.05	46.30	<0.001
GDP (current US dollars)	1.00	3.88	0.049
Year	1.00	65.35	<0.001
Spatial smooth: s(longitude, latitude)	23.40	228.35	<0.001

Table S11. Sensitivity analysis 9: GAM with country-level random effects

Variables	Estimated degrees of freedom	X2	P
Mean annual temperature (°C)	1.00	11.02	<0.001
Urban population percentage (%)	23.52	147.57	<0.001
Year	3.76	64.96	<0.001
Country (random effect)	60.22	693.07	<0.001

Table S12. Sensitivity analysis 10: GAM with COVID-19 period fixed effects

Variables	Estimated degrees of freedom	X2	P
**Smooth terms**			
Mean annual temperature (°C)	5.43	106.73	<0.001
Urban population percentage (%)	28.98	317	<0.001

GDP (current US dollars)	1.37	11.46	0.0016
**Period fixed effects**	**Coefficient**	**Std. Error**	**p-value**
Pandemic period (2020–2021)	−1.59	0.36	<0.001
Post-pandemic period (2022–2024)	−1.43	0.29	<0.001

Note: The pre-pandemic period (2004–2019) was used as the reference category for period fixed effects. Incidence rate ratios (IRRs) were derived by exponentiating the coefficients. The pandemic period corresponded to an IRR of approximately 0.20, and the post-pandemic period corresponded to an IRR of approximately 0.24.

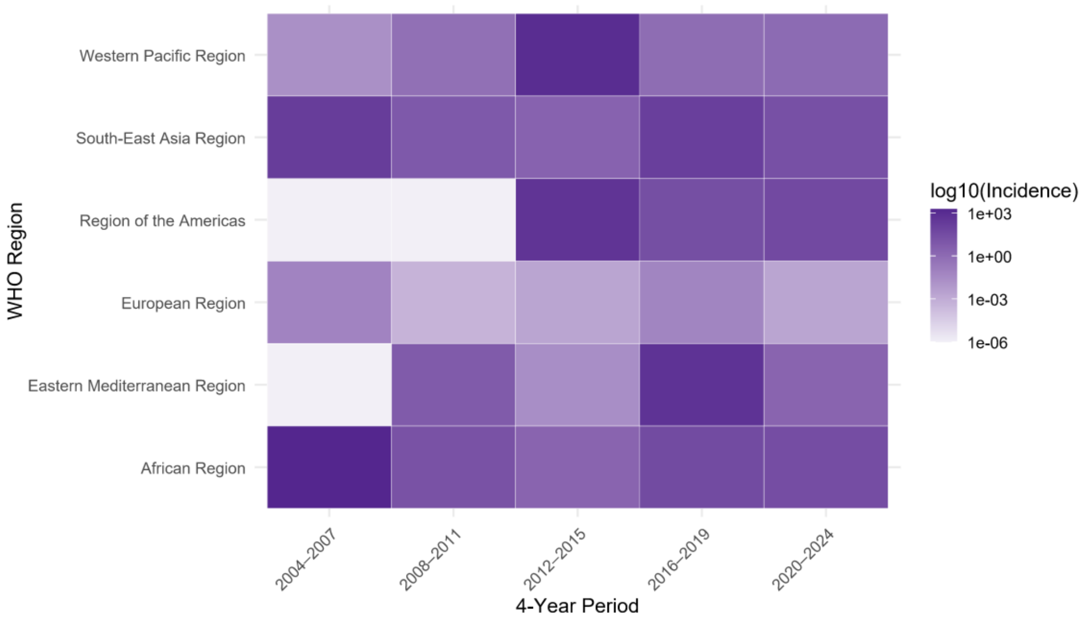

Figure S1. Average reported chikungunya incidence (per 100 000 population) by WHO region and four-year period (2004–2024).

Note: Values are log-transformed for visualisation; original zero values were replaced with 1 × 10−6 prior to transformation.

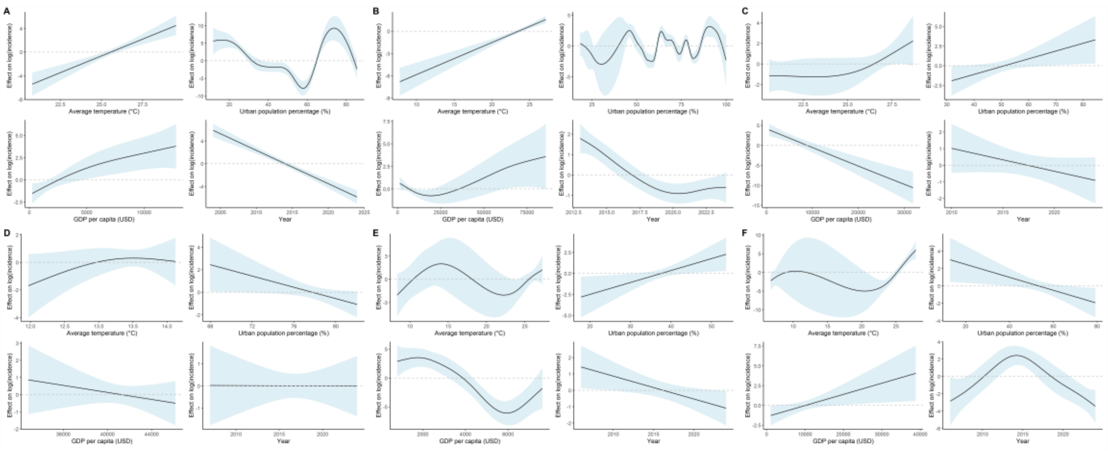

Figure S2. Partial Residual Plots by WHO Region Showing the Effects of Covariates on Chikungunya Incidence (Link Scale). (A) African Region, (B) Region of the Americas, (C)

Eastern Mediterranean Region, (D) European Region, (E) South-East Asia Region, (F) Western Pacific Region.

Note: Shaded areas represent 95% confidence intervals.

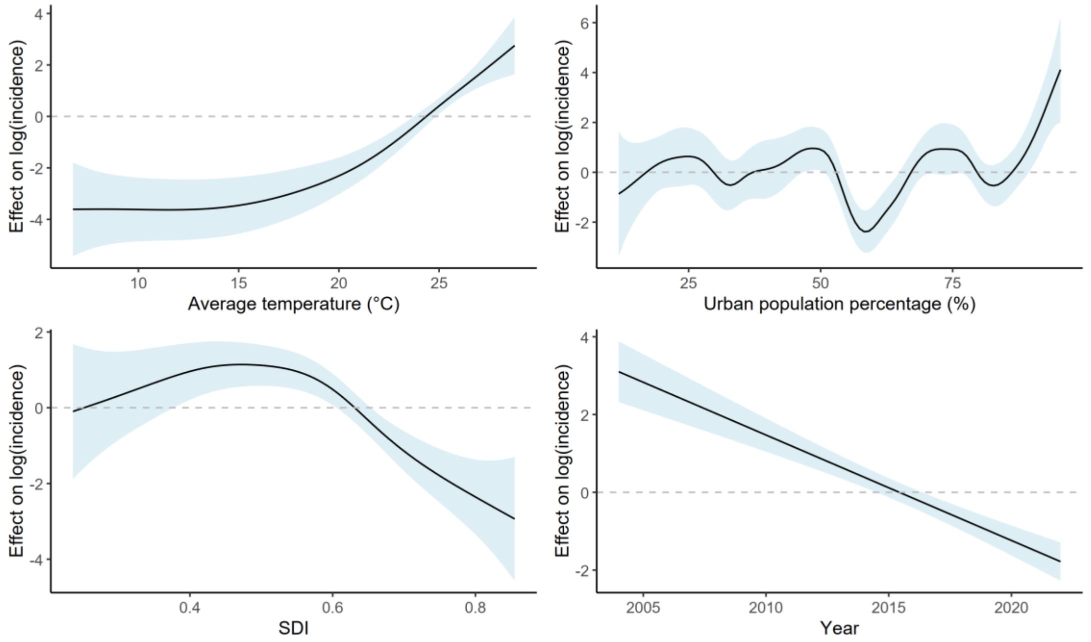

Figure S3. Partial Residual Plots from Sensitivity Analysis (Replacing GDP with SDI) Showing the Effects of Covariates on Chikungunya Incidence (Link Scale).

Note: Shaded areas represent 95% confidence intervals.

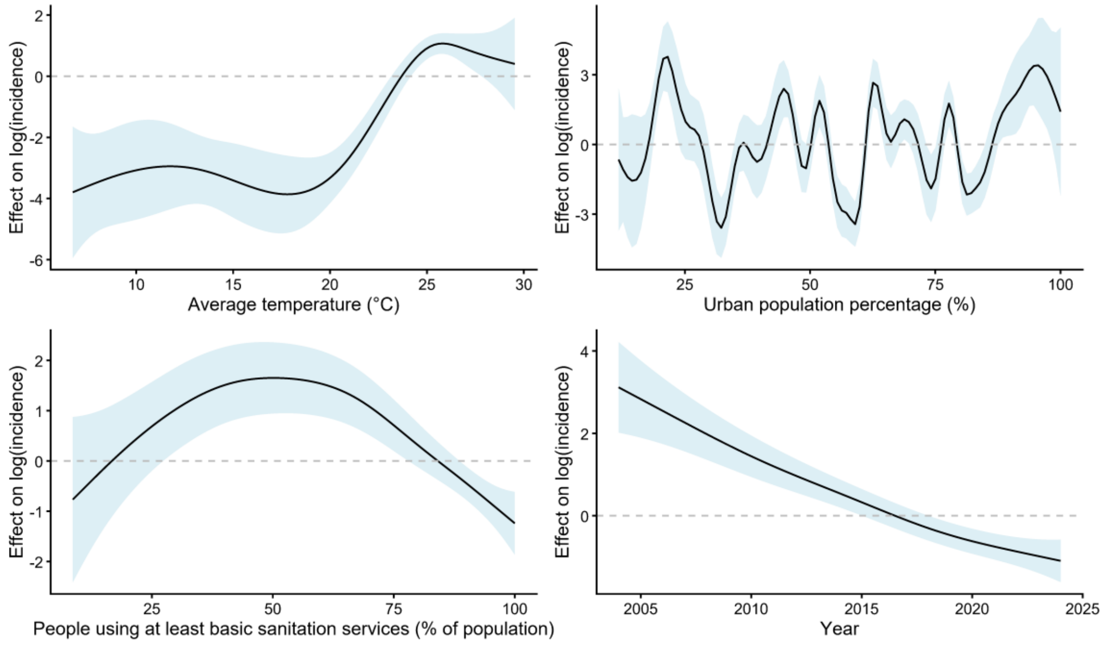

Figure S4. Partial Residual Plots from Sensitivity Analysis (Replacing GDP with people using at least basic sanitation services (% of population)) Showing the Effects of Covariates on Chikungunya Incidence (Link Scale).

Note: Shaded areas represent 95% confidence intervals.
